# Human Monkeypox: An Emerging Zoonosis

**DOI:** 10.7759/cureus.31736

**Published:** 2022-11-21

**Authors:** Janhvi Giradkar, Mahalaqua Nazli Khatib

**Affiliations:** 1 Physiology, Jawaharlal Nehru Medical College, Datta Meghe Institute of Medical Sciences, Wardha, IND; 2 Preventive Medicine, School of Epidemiology and Public Health, Jawaharlal Nehru Medical College, Datta Meghe Institute of Medical Sciences, Wardha, IND

**Keywords:** monkeypox, covid-19, immunity, pathogen, outbreak

## Abstract

Human monkeypox is caused by a zoonotic *Orthopoxvirus* that resembles smallpox. It is challenging to identify the illness from varicella and smallpox. The rapid spread of cases across countries has raised serious concerns among public health officials around the world, prompting accelerated investigations to identify the origins and causes of the rapid expansion of cases. When people come into contact with infected animals, they may unintentionally contract monkeypox. The monkeypox virus is transferred by direct exposure to lesions, respiratory droplets, body fluids, and contaminated objects like blood. Fever, rash, and lymph nodes frequent swelling are clinical signs of monkeypox, which can cause a multitude of health problems. The disease's worldwide significance is shown by the advent of outbreaks outside of Africa. To understand the constantly shifting epidemiology of this disease that is reemerging, monkeypox cases require improved monitoring and case identification. Before smallpox's eradication and the consequent absence of immunization attempts, vaccinia vaccination provided coincidental protection to the monkeypox virus; however, monkeypox gained therapeutic relevance. Additionally, given that rural Africa is where monkeypox cases are most common, it is possible that underreporting could result in an underestimate of the pathogen's potential impact. In recent months, cases of human monkeypox have alarmingly increased in nations where the illness is not prevalent. The current monkeypox outbreak, in light of the COVID-19 pandemic, represents a fresh threat. Clinicians need to be aware of this novel scenario, which differs from previous epidemics' scenarios. The transmission of monkeypox should be reduced by the development of efficient solutions by global health systems.

## Introduction and background

When Singaporean researchers sent monkeys to a Danish research institution, the monkeys became ill. Then in 1958, the monkeypox virus was first recognized and isolated. However, the virus was only spread throughout the Democratic Republic of the Congo by a toddler who was thought to have smallpox in 1970, making that the year of the first known human case [1]. The greatest animal reservoirs for the virus are rats, particularly huge pouched rats and squirrels that are hunted for food [2]. The Monkeypox virus (MPV) is a member of the genus *Orthopoxvirus* and its relatives *Poxviridae*. The cowpox, smallpox, and vaccinia viruses, as well as variola, are all members of the *Orthopoxvirus *genus. MPV is a virus with double-stranded DNA. The brick-shaped or oval structure of the poxviruses, which measure 200-400 nm, is well recognized [3].

## Review

Etiology

Viruses that have double-stranded RNA, known as *Poxviridae,* infect a diverse array of creatures, such as birds, reptiles, insects and mammals. The family is divided into two subfamilies:* Entomopoxvirinae* and *Chordopoxvirinae* (18 genera and 52 different species combined, containing 30 species across 4 genera). The *Chordopoxvirinae* subfamily, the *Poxviridae *family, and the *Orthopoxvirus* genus all contain the virus that causes monkeypox [4]. About 50% of the other genes are well conserved among poxviruses in vertebrates and are required for the viral reproduction, whereas the majority of the remaining genes are referred to be "accessory" genes, which play a major role in virus-host interactions [5].

Clinical features

**Figure 1:**
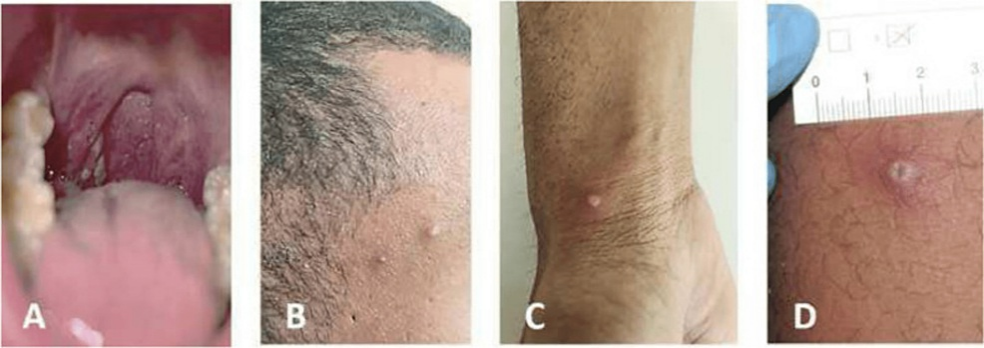
(A) Oral lesions that were evident at the patient's initial visit. (B-D) Vesicular and pustular lesions spread throughout the body. Source [5] Srivastava G, Srivastava G. Human Monkeypox Disease. Clin Dermatol. 2022 Aug 10:S0738-081X(22)00113-4. doi: 10.1016/j.clindermatol.2022.08.009. Epub ahead of print. PMID: 35963500; PMCID: PMC9364921.

The majority of the clinical features of human infection with monkeypox resemble those of smallpox (discrete ordinary type or modified type) [6]. Human monkeypox (MPX) is typically characterized by head and body aches, chills, sore throat, malaise, and exhaustion, lymph nodes that are enlarged and exhibit the characteristic skin rash with papules and vesicles before ultimately crusting over and recovering [7]. The incubation phase lasts from 5 to 21 days and typically lasts for 6 to 13 days. Although all ages are impacted, the median age has risen over time. More men than women are impacted [8]. Rashes of all sizes start to occur 1 to 5 days after the fever starts on the body, hands, legs, and feet after starting with the face. Beginning with vesicles (fluid-filled blisters), pustules, macules, and papules, the rash progresses through several stages until clearing up over time with crusts and scabs that fall off when the patient is healthy. The rash can be going through numerous stages at once (Figure 1). Around distinct lesions, regions of skin hyperpigmentation or erythema are frequently visible. Sometimes, detached scabs are much smaller than the initial lesions. There may also be pharyngeal, conjunctival, and vaginal mucosal inflammation [9]. Lymphadenopathy, which distinguishes human monkeypox as opposed to smallpox, is a significant characteristic. Lymphadenopathy develops in approximately 90% of MPV-infected patients, which can be unilateral, bilateral, or a combination of these, and impacts the inguinal, axillary, postauricular, cervical, or submandibular lymph nodes [10]. The most distressing symptoms reported by patients were extensive skin conditions, itching, uncomfortable pustules, and genital ulcers, which can all cause deformity. During admission, 11 of the 40 (27.5%) patients acquired anxiety and depression symptoms, necessitating psychological counseling [11].

There are two possible Monkeypox virus (MPXV) transmission modes: both transmissions between people and animals and people. Inter-human transmission has been linked to the groin and vaginal lesions, bronchial droplets, body fluids in contact with polluted surroundings or goods, an infected individual's skin damage, and sexual transmission from an infected person. Animals can transmit diseases to a humans directly by the intake of or direct contact with natural viral hosts. Furthermore, zoonotic transmission can occur when blood or body fluids are in contact with a person or vaccination through mucocutaneous of an infected animal's lesions [12]. To date, the majority of reported fatalities involved young children and human immunodeficiency virus (HIV) positive persons [13]. Human monkeypox virus (MPXV) has been classified into two clades: West Africa and the Congo Basin (CB). The latter has a history of a rise in morbidity, mortality, and transfer from person to person [14]. Human-to-human networks of transmission, however, have generally received less attention. According to a pooled estimate from a comprehensive analysis, household contacts who had not received the smallpox vaccine experienced secondary attacks at a rate of about 8% (interquartile range: 0%-11%). The therapeutic significance of persistent viremia and skin shedding is still unknown, and there is little knowledge of the in-vivo viral kinetics and infectivity [15]. Smallpox immunity declines coronavirus infection in 2019 (COVID-19). The present global spread of monkeypox virus infection in humans reflects changes in the virus's biology, human behavior or both. Prevention measures are loosened, international travel is resumed, and sexual contact at huge gatherings are resumed. As of yet, the dissemination has disproportionately afflicted homosexual and bisexual males and others who are males and have sex with men, indicating that sexual networks are amplifying the spread of the disease [16].

Virus host range

According to recent findings from virus isolation and serological surveys, primates are accidental hosts for MPXV, whereas some African rope squirrels, in particular, are sylvatic rodent species and may serve as reservoir hosts [17]. A very wide variety of species of mammals were found to be vulnerable to MPXV contamination in a lab or captive settings [18], including other rodent species like prairie dogs and ground squirrels, as well as rabbits, ant-eaters, and opossums. It is significant to remember that ground squirrels, which are typical in North American grasslands, are extremely vulnerable to MPXV [19]. Concerns have been raised about MPXV establishing reservoirs in North American rodents as a result of human spillovers to animals. The ability of MPXV to use multiple host species is thought to be important for its survival in nature [18]. E3L and K3L, two poxvirus host range genes are known to target Protein Kinase R (PKR), which has been determined to be an essential barrier for poxvirus replication. It was discovered that the PKR genes of primates have quickly evolved and show heterogeneity in K3L inhibition susceptibility [17].

2022 monkeypox clinical manifestation

Case studies from the 2022 epidemic depict that skin lesions usually do not have a prodrome and are usually quiet, and lesions on a specific body area location may be at various phases of growth, in contrast to the traditional monkeypox presentation. Additionally, the rash and enanthem may appear simultaneously with the constitutional symptoms. Bruises on the soles and palms, as well as lymphadenopathy are less frequent. Therefore, these characteristics may not be very useful in distinguishing monkeypox from other illnesses. Monkeypox can resemble some more prevalent illnesses. Because there are many possible diagnoses for monkeypox, prehospital doctors should have a low bar for applying monkeypox measures [20]. It may be difficult to manage affected people during the outbreak in 2022 because of the virus's ambiguous appearance. Because monkeypox can be contagious during a prodromal period that is subclinical or before the formation of rash, prehospital doctors should consider this condition when making identification in patients with exposure to dangerous elements. Reducing the spread of monkeypox contributes to reducing extra worldwide strains on the healthcare system. Physical segregation, symptom monitoring, and common hygiene practices are methods for reducing transmission. These methods may eliminate the requirement regarding the confinement of exposed healthcare professionals, conserving the capacity of the healthcare system [21]. Patients who seek medical attention might simply have gone blind from their contagious skin sores and prodromal signs. Prior exposure, viral clade, illness stage, vaccination status, and all affect the differential diagnosis. There are several dermatological disorders and sexually transmitted diseases (STDs) that monkeypox can resemble [22].

Epidemiology

In 2022 on August 4, the epidemic of monkeypox was declared by the Health and Human Services Department in the United States. On May 17, following the discovery of the country's first instance of monkeypox, improved case identification and reporting for monkeypox were introduced by the Centers for Disease Control and Prevention (CDC) and state health authorities. By July 27, in Puerto Rico, 43 states and the District of Columbia had reported 2,891 cases, and the CDC had formed for 1,195 (41%) case reports received of those cases through July 22 [23]. Monkeypox instances increased in nonendemic nations throughout Europe and North America in May 2022. On May 19, 2022, Canada reported its first occurrences in Montréal. The World Health Organization classified the real-time polymerase chain reaction (PCR) laboratory confirmation of the first three MPX cases was supplied by the National Institute of Public Health (Instituto Nacional de Sade Doutor Ricardo Jorge, INSA, IP) on May 17 and declared monkeypox outbreak as a pandemic on June 22, 2022 [24].

Upon the discovery of these initial cases, the animal health laboratory of the General Directorate of Food and Veterinary Medicine in Portugal was informed. According to our case definition, 96 instances in Portugal of MPX have been confirmed as of May 27, 2022. Forty-one of these instances contained information on the exposure and symptom onset dates. The initial instances in Portugal began exhibiting symptoms as soon as April 29, according to an epidemic curve, and cases were still being found after the analysis was complete (April 29, 2022, to May 23, 2022). Moreover, a graph of the epidemic demonstrates various exposure pathways, such as frequenting specific places like saunas that are used for sexual activity, visiting other nations (Spain, the United Kingdom, and Brazil), and interacting with individuals who are not Portuguese nationals [25].

There were 521 cases of monkeypox as of June 22, 2022, documented in Germany. The average age of the cases, which were all male, was 38. Nearly all of the 69% of cases in Berlin involved males having sex with men. According to the German case definition, 521 laboratory-confirmed MPX cases had been reported to local public health departments (LPHA) as of June 22, 2022, and electronically transmitted to the Robert Koch Institute (RKI) through state health departments. On May 20, 2022, the first instance was notified. The median age of the cases-all men between the ages of 20 and 67 was 38; the interquartile range (IQR) was 32 to 44. Four hundred and fifty-five instances had their hospitalization status stated, and 38 of those (8%) had their admission to a hospital [26]. A male less than 10 years old without any relevant medical history presented at an Amsterdam, Netherlands, pediatric emergency department (ER) at the end of June 2022 [27].

Argentina reported the first two South American MPXV instances on May 27, 2022. Two and nine days before case notification, respectively, both reported cases traveled to Spain, and the two MPXV partial genomes (942 base pairs), the first monkeypox sequences from Latin America, were made accessible. Probable monkeypox infections were reported in Brazil as of June 8, 2022, in the nations of Sao Paulo, Rio Grande do Sul, Mato Grosso do Sul, Santa Catarina, Ceará, and Rondônia [28]. A total of 29 cases that met the requirements of the WHO case definition as of June 6, 2022, were recorded in Italy, the cases' average age was 36 years, and 16 of the 18 individuals who were all but one of them male were admitted to having sex with other guys (range: 20-54 years). All of the patients had a rash when they first arrived, which was localized to the genital or perianal region in 18 of the 21 cases; in the cases where the distribution of the lesions was unknown, no rash was evident. Twelve out of the 22 instances for which there was information reported having a fever [29]. Four MPX cases had been recorded in India as of July 24, 2022; the first case had been reported on July 14. They were all men. The first three cases were from Kerala and had prior international travel, but the final instance was from Delhi and had no prior international travel [8].

Treatment

Most monkeypox sufferers recover on their own, without any medical intervention. Those with gastrointestinal issues should drink more water to prevent fluid loss from the symptoms (such as vomiting or diarrhea) and will need oral or intravenous rehydration [30]. Several antivirals have been licensed based on animal models for the treatment of smallpox, but they may also be beneficial in treating monkeypox infections. Human dose studies for these medications have been carried out, but their effectiveness has not been fully explored. The first antiviral licensed for the treatment of smallpox in adults and children weighing at least 3 kg is tecovirimat, also known as TPOXX or ST-246. It is regarded as the most successful course of action [31]. In patients with advanced illness, tecovirimat and Brincidofovir may be used in dual therapy. When VP37, the viral envelope protein, is repressed, the last stages of viral maturation and release from the infected cell are stopped, which inhibits the virus from propagating inside the infected host [32]. Although the effectiveness of this medication against monkeypox in people has not been investigated, research on animals shows that tecovirimat treatment increased survival from deadly monkeypox virus infections when compared to placebo treatment at various illness phases [31]. The side-effect profile in a larger safety investigation included 359 human volunteers and the tecovirimat of the placebo drug was nearly identical to that of tecovirimat [33].

In modest studies, tecovirimat and vaccinia immune globulin (VIG) was given to patients who had smallpox vaccine side effects like progressive vaccinia and eczema vaccination. The Emergency Access Investigational New Protocol, according to the CDC, tecovirimat can be used to treat infections caused by non-variola *Orthopoxvirus*, like monkeypox. For pediatric patients who are under 13 kg, the protocol also permits removing a capsule from an oral combining its contents in conjunction with liquid or soft food. The Strategic National Stockpile provides tecovirimat as an intravenous vial or an oral capsule formulation [31]. Brincidofovir, an oral version of the injectable Cidofovir may have a better safety profile than cidofovir, including less kidney damage [34].

These drugs inhibit viral DNA from being produced. The effectiveness of Brincidofovir against *Orthopoxvirus* infections has been demonstrated, despite the paucity of studies examining its usage in treating monkeypox infections in animal models. Cidofovir has been shown in vitro to be effective against lethal monkeypox virus infections in animals, even though there is limited clinical research on its efficiency against monkeypox in people. It is necessary to administer cidofovir together with probenecid treatment and intravenous normal saline. As Brincidofovir may result in elevations in transaminases and bilirubin levels in the blood, it is necessary to do both before and after treatment liver function tests. These treatments are accessible through an Emergency Use Authorization (EUA) [31]. For a list of various medicinal drugs (Table 1).

**Table 1:** Medications to be given during monkeypox virus infection treatment CrCl: Creatinine clearance, IV: Intravenous therapy, DNA: Deoxyribose nucleic acid

Reference	Treatments	Dosing	Mode of action	Use in specific populations
Rizk et al [31]	Tecovirimat	Adults: 600 mg twice daily for 14 days. Children : between 13 kg to 25 kg, 200 mg twice daily; between 25 kg to 40 kg, 400 mg twice daily for 14 days; above 40 kg, 600 mg twice daily for 14 days.	Inhibitor of the orthopoxvirus VP37 envelope wrapping protein.	Hepatic/renal adjustments are not necessary. Patients with significant renal impairment should not be given IV.
	Brincidofovir	Adults: weighing 48 kg, 200 mg twice daily; Adults and children: weighing between 10 kg to 48 kg, 4 mg/kg of the oral suspension twice weekly; Children: weighing less than 10 kg, 6 mg/kg of oral suspension twice weekly.	Cidofovir diphosphate is a phosphorylated active metabolite that preferentially inhibits orthopoxvirus DNA polymerase-mediated viral DNA synthesis.	Not advised to pregnant or breastfeeding women (a pregnancy test should be performed before therapy in women of childbearing potential). Perform liver function tests before and during treatment since brincidofovir can produce a rise in blood transaminases and bilirubin.
	Cidofovir	5 mg/kg once weekly for 2 weeks, followed by 5 mg/kg IV once every other week.	After cellular phosphorylation, inhibits orthopoxvirus DNA polymerase-mediated viral DNA synthesis.	It is required to alter the dose based on renal function: Serum creatinine levels greater than 1.5 mg/dL, CrCl levels greater than 55 mL/min, or urine protein levels greater than 100 mg/dL.

Diagnosis

Someone exhibiting the aforementioned symptoms may have monkeypox, particularly if they have traveled or had contact with people who have the disease. Using the polymerase chain reaction (PCR) test can confirm a case of monkeypox that has been suspected [35]. Because this disease's symptoms are still challenging to recognize and treat in low-income countries. Since this disease is endemic to certain regions, it presents a global challenge [6]. Serology, tissue immunohistochemistry, electron microscopy, molecular diagnostics, and viral isolation are some of the available laboratory techniques for diagnosis. They include additional molecular assays such as restriction-fragment-length polymorphism (RFLP), loop-mediated isothermal amplification (LAMP), recombinase polymerase amplification (RPA), and other methods. MPX can be identified with high sensitivity and specificity using real-time PCR (RT-PCR) testing on skin lesions, throat, blood, and urine samples. These tests aren't offered commercially and are pricey. After five and eight days of infection, immunoglobulin M (IgM) and immunoglobulin G (IgG) against MPX-specific may be found using an enzyme-linked immunosorbent assay (ELISA). The distinct pox viruses are not distinguished by these, which are genus-specific. IgG can also be positive as a result of past exposure or vaccination against smallpox. IgG is less focused than IgM. An *Orthopoxvirus* Bio Threat Alert point-of-care diagnostic test (Tetracore, Rockville) may quickly and accurately identify poxvirus antigens in samples collected from skin lesions. Although PCR is more sensitive, it is unable to identify MPX from other pox viruses, nonetheless effective in field situations [8]. The Indian government has published recommendations for diagnosing MPX patients. For the *Orthopoxvirus* genus-specific PCR, skin scrapings, ethylenediamine tetraacetic acid (EDTA) as samples, blood, serum urine, and nasopharyngeal/oropharyngeal swabs will be processed. If the results are positive, the samples will be processed for MPX-specific PCR [8]. 

## Conclusions

The disease burden from MPX will rise, and it has been deemed a global emergency. It is important to remember that several of the nations with verified cases of monkeypox during the epidemic in May 2022 do not have an endemic monkeypox population, and the patients had no travel ties to endemic regions. However, in view because of the devastation brought on by the COVID-19 epidemic, it is imperative to thoroughly investigate the public health consequences and pandemic potential of monkeypox. The drop in population immunity resulting from the end of smallpox immunization has created the conditions for the return of monkeypox. Monkeypox cases and median age are on the rise, and outbreaks have returned to various countries after a pause of 30 to 40 years, illustrative of this. The fact that cases are now appearing emphasizes the disease's potential for geographic spread outside of Africa and how important it is to everyone in the world. Healthcare personnel who treat sick patients are also apprehensive about the possibility of human transfer. Given the contemporary atmosphere of pandemic dangers, the significance of the public health crisis caused by monkeypox should not be overstated. International funding is necessary for better case detection and surveillance to better understand the epidemiology of this resurgent sickness, which is constantly changing.
